# Implementation of Function and Behavior Focused Care

**DOI:** 10.1093/geroni/igaa057.2809

**Published:** 2020-12-16

**Authors:** Barbara Resnick, Elizabeth Galik

**Affiliations:** 1 University of Maryland School of Nursing, Baltimore, Maryland, United States; 2 University of Maryland School of Nursing, Ellicott City, Maryland, United States

## Abstract

There are many challenges to engaging long term care residents with dementia in physical and functional activities. Resident factors include age, comorbidities, cognitive impairment, motivation, sedation and polypharmacy. Facility factors include the environment, policies and culture within the setting such as a focus on safety versus function. Commonly used non-evidence based interventions include discouraging residents from walking by repeatedly telling them to sit down, and limiting recreational activities to seated positions. De-implementation to remove inaccurate care practices and implementation approaches are needed to facilitate implementation of Function and Behavior Focused Care. This paper will describe the use of the Synthesis Model for the Process of De-Adoption and the Evidence Integration Triangle implementation strategy in the development of the Function and Behavior Focused Care intervention to alter behavior among staff and cognitively impaired residents and optimize function and physical activity in long term care.

